# Correcting facial asymmetry through guided plate assisted mandibular angle osteotomy

**DOI:** 10.3389/fsurg.2024.1391231

**Published:** 2024-08-01

**Authors:** Wenqing Han, Zhang Yichi, Byeong Seop Kim, Mengzhe Sun, Gang Chai

**Affiliations:** ^1^ Shanghai Ninth People’s Hospital, School of Medicine, Shanghai Jiao Tong University, Shanghai, China; ^2^ Department of Plastic and Reconstructive Surgery, Shanghai Ninth People’s Hospital, School of Medicine, Shanghai Jiao Tong University, Shanghai, China

**Keywords:** mandible, osteotomy, computer-assisted surgery, patient satisfaction, facial asymmetry

## Abstract

**Background:**

Asian women prefer a smooth and narrowed mandibular appearance. The purpose of the retrospective cohort study is to evaluate guide plate-assisted mandibular angle ostectomy (MAO) in improving mandibular symmetry for Asian female patients with mandibular angle hypertrophy (MAH) with normal occlusal relationship.

**Methods:**

We retrospectively examined 11 patients with asymmetry MAH with normal occlusal relationship who received MAO at Shanghai Ninth People's Hospital between September, 2020, and January, 2022. Preoperative plans were designed based on CT data and executed using metal guide plate during the operation. Preoperative and one-week postoperative CT scans were used to assess measurements including Height_Go, Divergence_Go, ∠ZyZy-GoGo, and osteotomy volume, to evaluate symmetry. For precision, compare the postoperative CT with the preoperative design, assessing osteotomy distance, angle, and volume error. Patient satisfacation was evaluated with Likert Scale in 6-month follow-up. Secondary lipofilling procedures were given as appropriate. Statistical analysis was performed using paired t-tests in SPSS.

**Results:**

The mean age of the 11 patients was 28.5 years (range 23–34 years). 2 of these underwent lipofilling procedures. No complications were observed during the following-up. Postoperative results were not statistically different from the design, demonstrating a precision of within 2 mm. Height_Go disparity within 5 mm get corrected notably, reducing asymmetry from 15.09% preoperatively to 2.74% postoperatively. Patients satisfaction was rated at 4.5 out of 5 in 6 month follow-up.

**Conclusions:**

Guide plate-assisted mandibular angle osteotomies achieve effective and precise surgery. This approach demonstrates a safe option for correction for mandibular asymmetry, achieving patient satisfaction.

## 1 Introduction

Given the cultural emphasis on slender facial contours, particularly in Asian aesthetics, achieving a smoother and narrower low face appearance is typically preferred (1). Mandibular angle osteotomy (MAO) is one of the most common surgery to reduce the lower face width, especially in patients with asymmetry mandibular angle hypertrophy (MAH) (2). However, the intraoral approach for MAO suffers from a restricted surgical field, challenging the surgeon's ability to ensure symmetrical adjustments (3). In addition, Mandibular asymmetry often involves variable positioning of the inferior alveolar nerve, significantly raising the risk of nerve damage and excessive bleeding (4).

While traditional surgery outcomes rely on the subjective experience of the surgeon, presently computer-aided design (CAD) became essential tools for achieving precise symmetrical results (5). Binbin Ying utilized CAD to design a multi-stage osteotomy plan and performed the surgery based on intraoperative anatomical landmarks (6). Furthermore, intraoperative guide positioning enhances the precision of surgery. Lee et al. used a self-curing resin guide osteotomy (7), but its stability was compromised under high-speed drilling and localized high temperatures. For a safer intraoperative positioning option, our team has achieved good results using metal guide plate in patients with simple MAH (8), but has not yet evaluated the effect of symmetric correction.

The purpose of this study is to evaluate the effectiveness, precision, and symmetry outcomes of the guided plate-assisted MAO procedure. This was achieved by comparing preoperative, design, and postoperative measurements using three-dimensional CT scans in an objective and quantitative manner. The focus is specifically on assessing the utility and benefits of the guided plate in improving surgical outcomes.

## 2 Methods

### 2.1 Patients

This retrospective study analyzed female patients with mandibular asymmetry defined as a difference in mandible ramus height (MRH) exceeded 3 mm, who underwent guide plate-assisted MAO at the Department of Plastic Surgery, Shanghai Ninth People's Hospital, between September, 2020 and January, 2022. These patients had no history of malocclusion or orthodontic treatments. The inclusion criteria required the CT imaging data preoperatively and one week after osteotomy, as well as standardized clinical photographs preoperatively and six months postoperatively. Informed consents were obtained from all patients before their participation.

### 2.2 Procedure

#### 2.2.1 CT imaging and preoperative design

The CT imaging protocols followed the requirements outlined in the previous study (9). Data were stored in Digital Imaging and Communications in Medicine (DICOM) format and transferred to Mimics software (version 18.0, Masterialise, Leuven, Belgium). The mandible was segmented using the threshold segmentation tool and the region growth tool. The bilateral mandibular nerve canals were drawn manually. The 3D model of the mandible was reconstructed after checking multi-view labeling. The design was standardized on the side with the lower mandibular nerve canal. The highest starting point of the osteotomy was located at the intersection of the occlusal plane and the posterior margin of the mandibular ramus. The end point of the osteotomy plane was located at the lower margin of the body of the mandible, beneath the mental foramen. The angle between the osteotomy plane and the mandibular outer plate plane was 50 degrees. Considering the measurement and operational errors, the minimum distance between the osteotomy plane and the mandibular nerve canal was clinically set to be >3 mm. If it could not achieve the standard, the starting point of osteotomy was adjusted downward to satisfy the minimum nerve distance. This design was defined as the maximum osteotomy plan and thoroughly communicated to the patient. The ultimate design should not be less than 50% volume of the maximum one, considering surgical outcomes. It was recommended to choose the maximum osteotomy volume whenever possible to allow space for subsequent lipofilling. The guide plate was manufactured as previously described (9).

#### 2.2.2 Guide plate-assisted MAO

**Figure 1:**
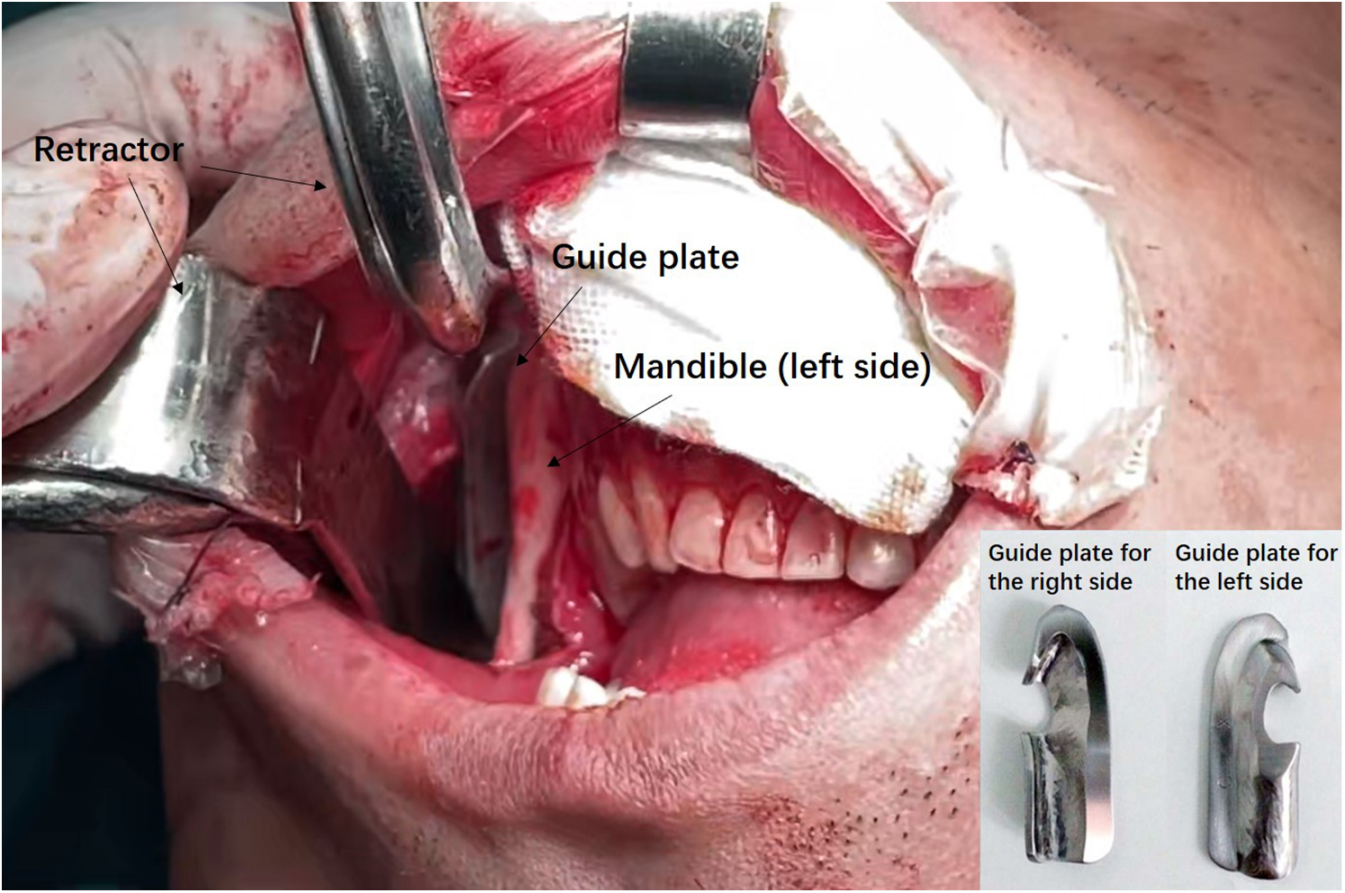
Intraoperative photograph of the guide plate-assisted MAO. The guide plated for the left side was mounted on the patient's mandible angle region, on which the saw blade was relying to perform ostectomy.

General anesthesia and transnasal tracheal intubation was performed to ensure a patent airway, followed by cleansing of the facial and oral areas using an iodine solution to keep the surgical area sterile. Local infiltration of the surgical site was performed using a solution containing 1/200,000 epinephrine to provide hemostasis effect. Subsequently, a mucosal incision was made at least 1 cm above the lateral labial sulcus to expose the mandibular branch and mandibular body. The surgical approach involved the submental and mandibular angle regions. Upon completion of the incision, subperiosteal dissection was performed. The guide plate was exactly positioned in alignment with the mandibular angle. Osteotomy was carried out along the upper edge of the guide plate (Figure 1). At the end of the procedure, intraoral incision was sutured and external dressings with compression were performed to prevent hematoma formation. Special attention was given to incision cleaning, oral rinsing, and maintenance a liquid diet for two weeks. CT was performed one week after surgery, while a follow-up visit was performed 6 months postoperatively to assess subjective satisfaction. Lipofilling surgery was considered as needed to achieve precise correction of the desired aesthetic outcome.

### 2.3 Objective measurements

**Figure 2:**
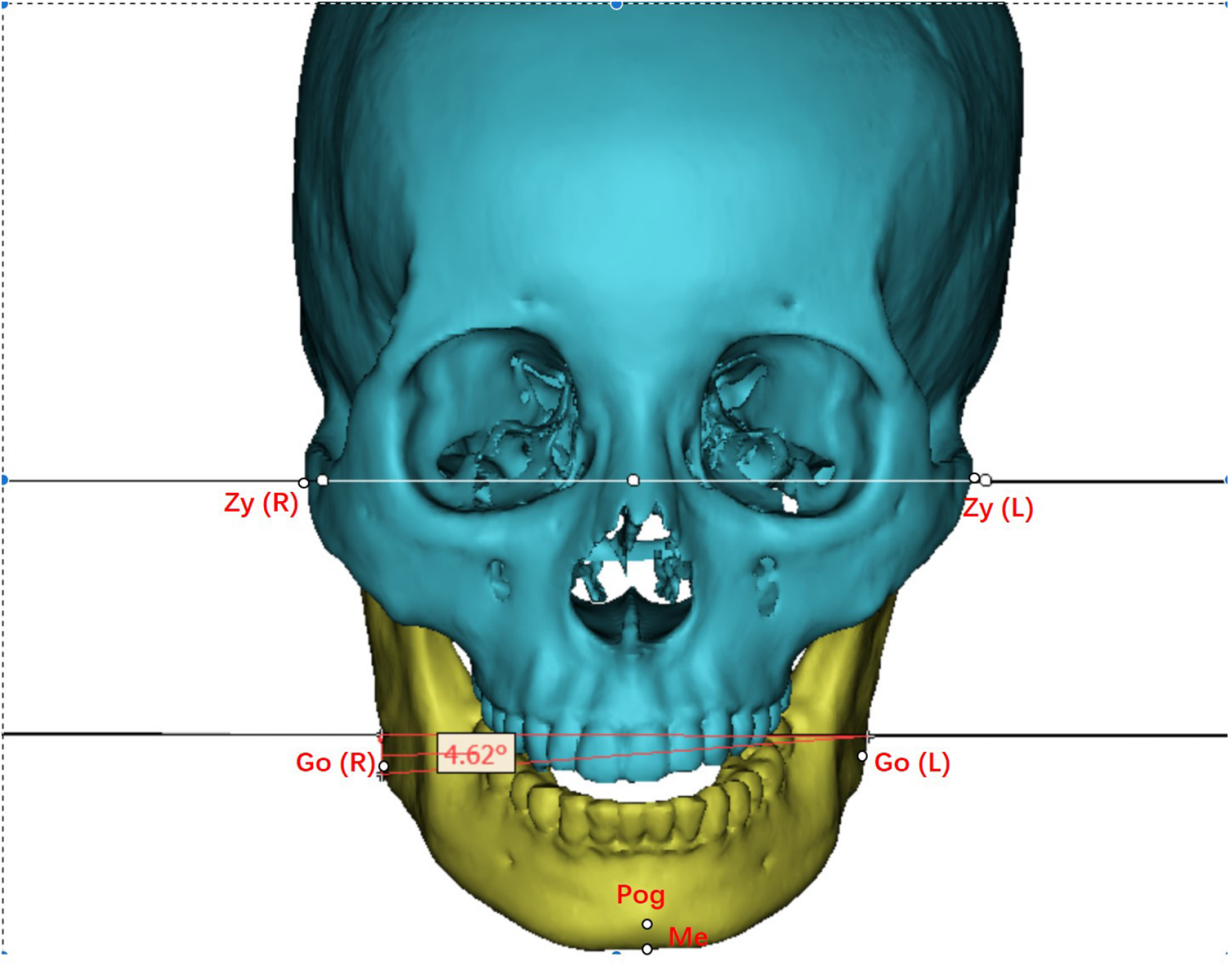
Measurement diagram—this figure demonstrates the methodology for measuring ∠ZyZy–GoGo (degrees).

**Figure 3:**
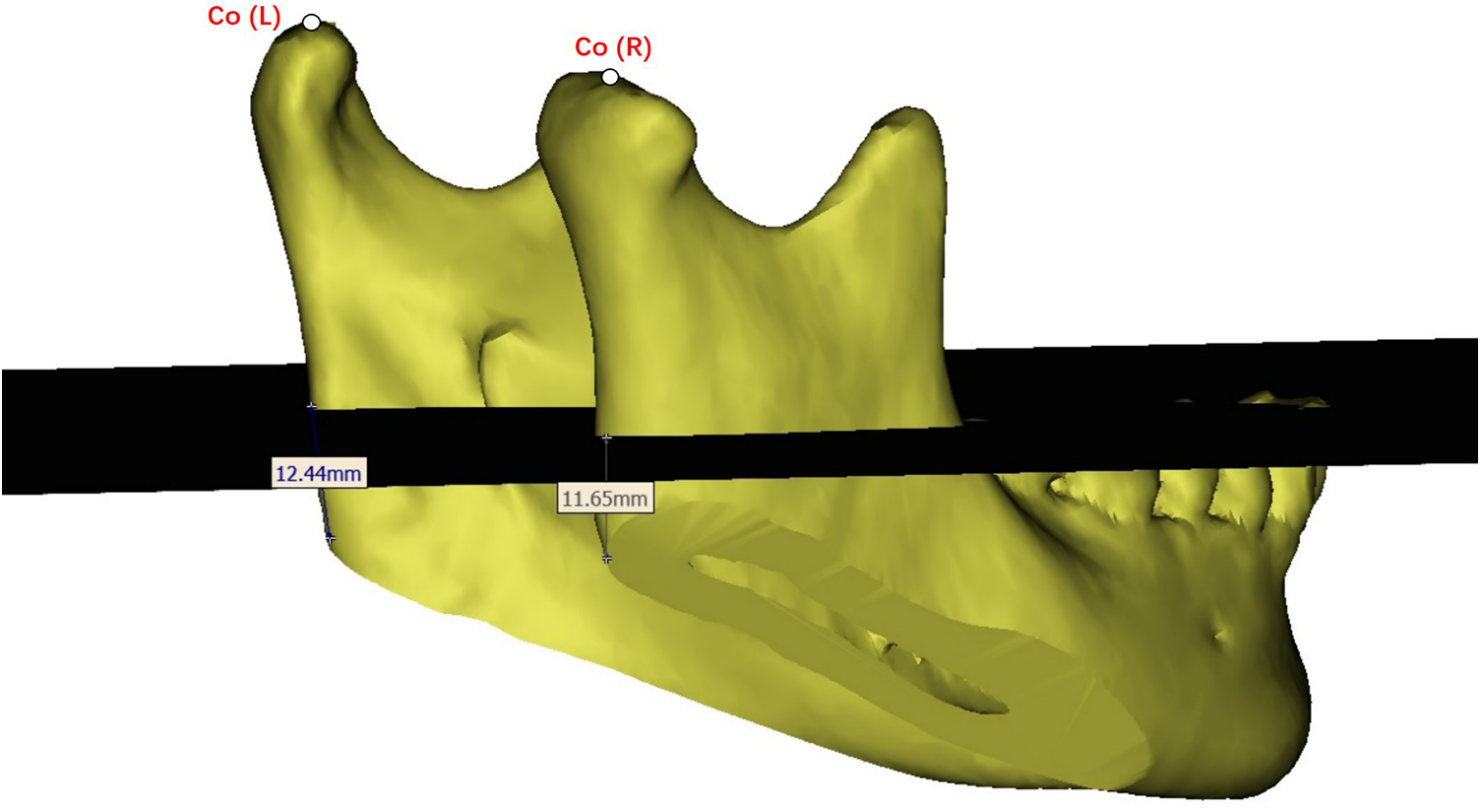
Height_Go (mm) illustrates the measurement of the distance from the gonion, the most outward point on the angle of the mandible, to the occlusal plane.

Preoperative design, and postoperative CT files were evaluated retrospectively. Landmarks included key points such as the mandibular angle point (Go), the anterior chin point (Me), and the condylar (Co) point. Measurements included Co-Go-Me mandibular angle, Co-Go mandibular ramus, Go-Me mandibular body length, HeGo (Height_Go) mandibular angle point to occlusal plane distance (Figure 2), DiGo (Divergence_Go) to median sagittal plane distance, and Go-Go gonial width. ∠ZyZy-GoGo (°) indicated mandibular deflection angle (Figure 3). At the same time, we measured and calculated the left-right difference in osteotomy volume, and the left-right difference in hemi-mandibular volume (Table 1). The symmetry was compared using the asymmetry calculation formula (Asymmetry = ABS[(R − L)/(R + L)]*2*100%).

**Table 1:** Landmarks and measurements definitions.

Landmark	Definitions	Measurements	Definitions
Pogonion (Pog)	The most anterior projecting point in the midline on the chin.	Zy–Zy	Middle facial breadth
Menton (Me)	The lowest point on the intersection between the mid-sagittal plane and the chin in Frankfurt horizontal plane	Go–Go	Lower facical breadth
Zygion (zy)	Most lateral point of the zygomatic arch	Me–Go	The length of the mandibular body
Gonion (Go)	Most prominent point of mandibular angle	HeGo (Height_Go)	The vertical distance from the gonion to the occlusion plane
Condylion (Co)	Most superior point on the mandibular condyle	∠Co–Go–Me	The mandibular angle
Occlusal plane	Mesial contact point of the lower central incisors to the distobuccal cusps of the last molars on both sides, represents an imaginary plane	Co–Go	The mandibular ramus
		DiGo (Divergence_Go)	The horizontal distance from the gonion to the midplane delineated by Nasion, Anterior nasal spine (ANS), and Basion.
		∠ZyZy-GoGo	The angle between the widest line of the midface and the widest line of the lower face
		Hemi_mandibular volume	Mandible volume divided by the mid-sagittal plane
		Osteotomy volume	The amount of bone that is removed or altered in position during an osteotomy procedure

### 2.4 Statistics

Data were analyzed using SPSS 22.0 software (SPSS, Chicago, USA). To evaluate the reliability of the measurements, both inter-examiner and intra-examiner reliability were analyzed. Two independent researchers conducted the same measurements independently. The same researchers performed repeated measurements at an interval of two weeks. The inter-examiner and intra-examiner reliability were assessed using the intra-class correlation coefficient (ICC). An ICC ranging from 0.81 to 1.00 indicates almost perfect agreement. The mean values were then calculated for further analysis.

Descriptive statistics, including mean and standard deviation, were used for data conforming to a normal distribution, while median was utilized for non-normally distributed data. Preoperative and postoperative CT measurements were analyzed through paired *t*-tests to evaluate the efficacy of the procedure. Postoperative and design CT data were examined to assess the procedure's precision. Additionally, symmetry calculations were performed. Statistical significance was set at *P* values less than 0.05 for all analyses.

## 3 Results

### 3.1 Demographic data

The mean age of the 11 patients in this study was 28.5 years (range 23–34 years). Of these, 2 (18.2%) underwent fat grafting; no complications were observed. Mean satisfaction with postoperative results amounted to 4.5 out of 5.

### 3.2 Measurements

**Figure 4:**
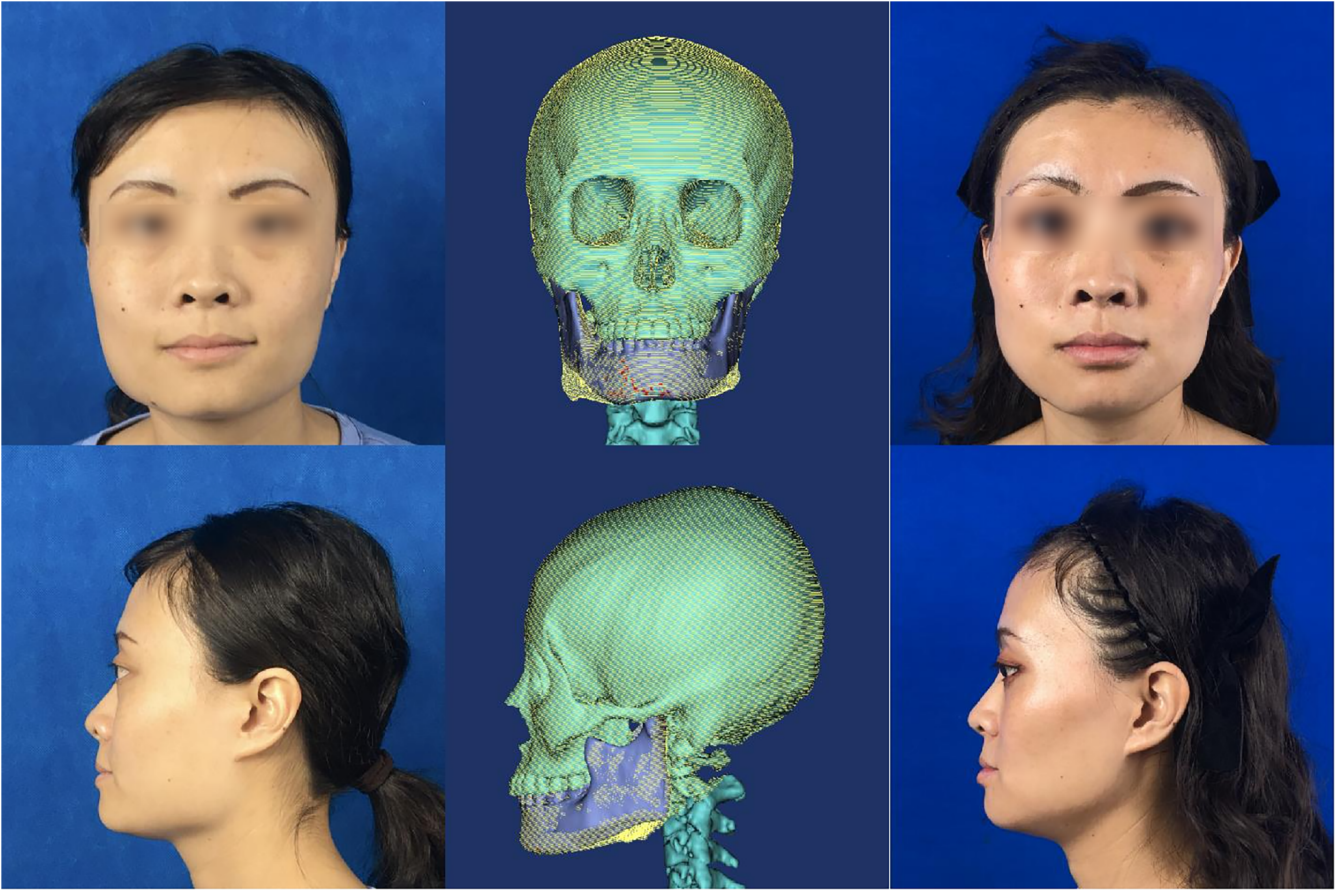
Typical clinical standard photos—displays a patient before and after undergoing guide plate-assisted mandibular angle osteotomy without lipofilling, highlighting high postoperative satisfaction.

Eleven patients underwent successful guided MAO without any complications (Figure 4). The intra-examiner reliability was assessed individually for both researchers. The mean values from each researcher were then used to analyze inter-examiner reliability. For all the measurements: the intra-examiner reliability for Researcher 1 (ICC range: 0.934–0.994) and Researcher 2 (ICC range: 0.956–0.990) was high, as was the inter-examiner reliability (ICC range: 0.945–0.995). All measurements demonstrated strong reliability.

In terms of effective, the measure Height_Go (mandibular height) showed a large change in asymmetry decreasing from 15.09% (preoperative) to 2.74% (postoperative). Bilateral Height_Go difference *p*-value of preoperative and postoperative paired *T*-test was <0.0001, showing a significant difference; the *p*-value of surgical planning and actual postoperative paired *T*-test was 0.8868, showing no significant difference. Meanwhile, ∠ZyZy-GoGo (°) decreased from 6.08° ± 2.19° to 1.40° ± 1.61°, indicating an effective correction of the lower face deviation. The *p*-value of ∠ZyZy-GoGo in preoperative and postoperative paired *T*-tests was 0.0004, with a significant difference; the *p*-value of surgical planning and actual paired *T*-test was 0.4644, with no significant difference.

It could be seen that the preoperative asymmetry of the patient was corrected after surgical treatment, and the execution of the surgical plan was accurate. The preoperative asymmetry indices for Me–Go, DiGo, Co–Go–Me (°), and hemi-mandibular volume were 1.16% ± 7.67%, 1.44% ± 1.91%, −1.01% ± 4.04%, and 1.36% ± 2.21%, respectively (Table 2). These indices were not sensitive and limiting their precision in reflecting surgical outcomes. No significant difference was found in *T*-test on other indexes.

**Table 2:** Preoperative, designed, and postoperative measurements for mandibular asymmetry.

Measurements	Preoperative		Designed		Postoperative	
	Dominant side/recessive side	Asymmetry%	Dominant side/recessive side	Asymmetry%	Dominant side/recessive side	Asymmetry%
Height_Go (mm)	29.41 ± 4.49/25.31 ± 3.08	15.09% ± 4.88%	14.12 ± 3.45/13.62 ± 2.41	1.70% ± 10.31%	14.50 ± 3.08/13.98 ± 2.93	2.74% ± 6.92%
Co–Go (mm)	74.22 ± 19.36/69.67 ± 18.38	6.40% ± 1.46%	57.61 ± 21.00/57.87 ± 19.19	−1.08% ± 4.20%	61.00 ± 22.87/60.76 ± 21.30	1.71% ± 3.62%
Me–Go (mm)	95.45 ± 7.89/94.20 ± 5.45	1.16% ± 7.67%	100.64 ± 3.57/100.52 ± 3.78	0.12% ± 3.38%	100.50 ± 4.06/100.28 ± 3.57	0.21% ± 5.52%
DiGo (mm)	51.64 ± 4.34/50.88 ± 3.97	1.44% ± 1.91%	49.41 ± 3.67/49.11 ± 3.63	0.62% ± 4.49%	49.36 ± 3.61/49.26 ± 4.35	0.32% ± 5.13%
HemiVolume (mm^3^)	45,442.83 ± 6,235.00/44,879.28 ± 6,450.10	1.36% ± 2.21%	40,762.33 ± 5,041.28/40,640.82 ± 5,317.48	0.39% ± 1.99%	40,147.56 ± 5,741.49/40,025.95 ± 6,036.16	0.41% ± 3.35%
∠Co–Go–Me (°)	99.11 ± 12.63/99.99 ± 11.89	−1.01% ± 4.04%	102.28 ± 12.01/103.80 ± 12.03	−1.50% ± 2.77%	102.72 ± 13.24/103.11 ± 12.92	−0.43% ± 2.61%
Me–Go (mm)	95.45 ± 7.89/94.20 ± 5.45	1.16% ± 7.67%	100.64 ± 3.57/100.52 ± 3.78	0.12% ± 3.38%	100.50 ± 4.06/100.28 ± 3.57	0.21% ± 5.52%
∠ZyZy–GoGo (°)	6.08° ± 2.19°		0.93° ± 1.09°		1.40° ± 1.61°	

In terms of precision, the actual postoperative CT was compared with the preoperative design. The average distance error of osteotomy was 1.66 ± 1.07 mm, the maximum distance error of osteotomy was 2.33 ± 1.19 mm, the angle error of osteotomy plane was 11.52 ± 2.69°, the volume error of osteotomy was 2.68 ± 0.955 cm^3^, and the mean value of the difference in the spacing of the angle of the mandibular angle was 1.60 ± 1.64 mm. These results indicate that the guide plate facilitated precise mandibular angle osteotomy and positioning, aligning with clinical requirements (Table 3).

**Table 3:** Comparison of actual postoperative CT with preoperative design for guide plate-assisted mandibular angle osteotomy errors.

Case number	Average osteotomy distance error (mm)	Maximum osteotomy distance error (mm)	Osteotomy plane angle error (°)	Osteotomy volume error (mm^3^)	Difference in mandibular angle width (mm)
1	0.66	1.28	12.02	2.24	0.82
2	3.63	4.10	13.63	2.44	1.44
3	2.42	2.88	10.14	2.23	3.48
4	3.26	4.26	17.42	2.98	0.59
5	0.54	0.82	9.80	2.68	0.6
6	1.77	3.17	11.18	3.35	1.54
7	2.08	2.96	7.67	4.33	0.91
8	0.89	1.60	13.59	4.18	0.36
9	0.71	1.36	12.08	1.58	5.83
10	1.05	1.54	8.91	2.12	0.86
11	1.23	1.67	10.28	1.39	1.19
Mean ± Standard deviation	1.66 ± 1.07	2.33 ± 1.19	11.52 ± 2.69	2.68 ± 0.955	1.60 ± 1.64

## 4 Discussion

In clinical practice, facial asymmetry is a common complaint, usually due to skeletal imbalance (10). The etiology of mandibular asymmetry is diverse and may be due to congenital factors, trauma, medical factors, or tumor treatment (11). Previous treatment methods include bone implantation, osteotomy, and lipofilling (12). In patients with mandibular hypertrophy combined with unilateral mandibular asymmetry, achieving a smoother and narrower low face appearance is typically preferred. We performed MAO using personalized guide plate, incorporating lipofilling as required. Based on 3d CT evaluation and subjective satisfaction assessment, the results demonstrate that this approach has achieved satisfactory clinical outcomes.

The intraoral incision approach has been widely used (13) since 1951 when Converse first reported mandibuloplasty under an intraoral incision (14). Kim and Park (4) resected mandibular angle by combining an intra- and extra-oral approach. Yoon Joo Lee reported 42 cases of MAO under a retroauricular approach. Considering the facial scarring, the intraoral incision remains the primary choice. There are various osteotomy surgical techniques, including straight line osteotomy, curve osteotomy and multi-stage osteotomy osteotomy (15–17). Straight line osteotomy aligns with current esthetic preference for a clear jaw line. The use of the reciprocating saw in this procedure is notable for its high efficiency in minimizing intraoperative exposure, while also presents relatively high in neurovascular damage (18). The use of CAD/CAM in asymmetric maxillofacial skeletal surgery was applied to address these concerns (8, 19–21).

In terms of CAD, studies development from manual approaches to algorithmic intelligent design. In 2021, Xiao-Yan Mao (6) used digital technology to automate the planning of mandibular angle curve osteotomy surgery in 25 female patients with MAH. Additionally, our research team proposed an efficient MAO program based on convolutional neural networks (CNNs) significantly reducing the need for manual intervention (22). However, the algorithm did not incorporate specific parameter adjustments for asymmetric individuals. Particular attention should be given to the imbalanced inferior alveolar nerve to ensure safe osteotomies. It is worth mentioning that optimizing the endpoint of the osteotomy design (under the mental foramen) not only prevented the appearance of a second mandibular angle but also provided more thorough correction for asymmetry of body of mandible.

In terms of CAM, in 2021, David M. Straughan (23) reported CAM allograft implants to repair mandibular asymmetry in 123 cases (of which 75% were males). In 2014, Matthew R (24), retrospectively studied 21 maxillofacial surgical procedures following lipofillings in 21 patients and showed that lipofilling improved the soft tissue contour covering repositioned bone or allograft implants (25). However, few Western studies have reported on MAO, possibly due to different aesthetic preferences.

Our results suggest that the precision of MAO assisted by metal guide plate was within 2 mm in terms of ostectomy plane error, meeting the requirements in craniomaxillofacial surgery. Asymmetry correction was deemed satisfactory. According to objective measurements, discrepancies such as MRH difference within 4–5 mm, mandibular deviation within 5°, an volume disparities within 0.45 cm^3^ could be successfully corrected. Additionally, based on the patient's satisfaction, the surgery remains effective during following up.

However, addressing mandibular asymmetry is complex, involving three-dimensional rotations that may be hard to fully corrected through hard tissue changes alone. Factors like Edema, differences in the healing process, and muscle reattachment may affect the final shape of the soft tissue. Therefore, follow-up is clinically scheduled after 6 months of mandibular surgery. Lipofilling procedures are selectively performed, aiming to precisely correct any remaining irregularities or asymmetries, offering patients additional opportunities to refine their mandibular asymmetry. Another less invasive choice to enhance facial contours and correct asymmetries is facial implant. Made from biocompatible materials, these implants can be customized to the patient's needs and provide significant aesthetic improvements with shorter recovery times (26, 27). However, implants come with risks such as displacement, infection, and potential need for future revisions (28). They may also not address underlying bone hypertrophy as effectively as osteotomies. While facial implants are valuable for facial contouring, the precision and effectiveness of guided plate-assisted MAO might make it a superior option for correcting mandibular asymmetry due to hypertrophy. Future studies could explore the combined use of implants and osteotomies to further enhance aesthetic outcomes (29).

In summary, we retrospectively investigated the benefits of high-precision osteotomies in patients with asymmetry MAH. However, the small number of subjects and the lack of randomization and controls limited this study. Limitations also include the lack of a quantitative way to evaluate soft tissue symmetry. Follow-up studies could incorporate three-dimensional evaluation data such as stereolithography to quantitatively assess soft tissue changes.

## 5 Conclusion

Guide-assisted precision mandible surgery, with optional lipofilling, offers patients the customizable opportunity to precisely improve mandible asymmetry. However, while the effectiveness of guide-assisted precision mandible surgery is acknowledged, this paper alone cannot confirm the efficacy of optional lipofilling. Further studies are required to explore the specific role and long-term outcomes of lipofilling in conjunction with mandible asymmetry correction.

## Data Availability

The raw data supporting the conclusions of this article will be made available by the authors, without undue reservation.

## References

[1] Satoh K. Mandibular contouring surgery by angular contouring combined with genioplasty in orientals. Plast Reconstr Surg. (1998) 101(2):461–72. 10.1097/00006534-199802000-00035 9462783

[2] Cui J Zhu S Hu J Li J Luo E. The effect of different reduction mandibuloplasty types on lower face width and morphology. Aesthetic Plast Surg. (2008) 32(4):593–8. 10.1007/s00266-007-9064-z 18034199

[3] Robson MC. An easy access incision for the removal of some intraoral malignant tumors. Plast Reconstr Surg. (1979) 64(6):834–5. 10.1097/00006534-197912000-00025 515237

[4] Kim Y Park B. Resection of the prominent mandible angle with intraoral and external approach. Aesthetic Plast Surg. (2003) 27(1):38–42; discussion 43. 10.1007/s00266-002-2078-7 12632199

[5] Zinser MJ Mischkowski RA Sailer HF Zöller JE. Computer-assisted orthognathic surgery: feasibility study using multiple CAD/CAM surgical splints. Oral Surg Oral Med Oral Pathol Oral Radiol. (2012) 113(5):673–87. 10.1016/j.oooo.2011.11.009 22668627

[6] Mao XY Fu X Niu F Chen Y Jin Q Qiao J Computer-assisted mandibular curved osteotomy: an automatic method to design the new aesthetic gonion and osteotomy line. J Plast Reconstr Aesthet Surg. (2021) 74(10):2622–8. 10.1016/j.bjps.2021.03.067 33952433

[7] Lee WS Lee DH Lee KB. Evaluation of internal fit of interim crown fabricated with CAD/CAM milling and 3D printing system. J Adv Prosthodont. (2017) 9(4):265–70. 10.4047/jap.2017.9.4.265 28874993 PMC5582092

[8] Lin L Han W Sun M Kim BS Chen X Aung ZM Current practices for esthetic facial bone contouring surgery in asians. Clin Plast Surg. (2023) 50(1):71–80. 10.1016/j.cps.2022.08.002 36396263

[9] Zhu M Liu F Zhou C Lin L Zhang Y Chai G Does intraoperative navigation improve the accuracy of mandibular angle osteotomy: comparison between augmented reality navigation, individualised templates and free-hand techniques. J Plast Reconstr Aesthet Surg. (2018) 71(8):1188–95. 10.1016/j.bjps.2018.03.018 29729839

[10] Erickson GE Waite DE. Mandibular asymmetry. J Am Dent Assoc. (1974) 89(6):1369–73. 10.14219/jada.archive.1974.0596 4529987

[11] Solem RC Ruellas A Ricks-Oddie JL Kelly K Oberoi S Lee J Congenital and acquired mandibular asymmetry: mapping growth and remodeling in 3 dimensions. Am J Orthod Dentofacial Orthop. (2016) 150(2):238–51. 10.1016/j.ajodo.2016.02.015 27476356 PMC5048942

[12] Choi JW Park H Kwon SM Lee JY. Surgery-first orthognathic approach for the correction of facial asymmetry. J Craniomaxillofac Surg. (2021) 49(6):435–42. 10.1016/j.jcms.2021.04.005 33934974

[13] Baek SM Baek RM Shin MS. Refinement in aesthetic contouring of the prominent mandibular angle. Aesthetic Plast Surg. (1994) 18(3):283–9. 10.1007/BF00449796 7976763

[14] Converse JM. Deformities of the jaw. In: Converse JM, editor. Reconstructive Plastic Surgery. Philadephia: WB Saunders (1977). p. 1406–8.

[15] Ying B Wu S Yan S Hu J. Intraoral multistage mandibular angle ostectomy: 10 years’ experience in mandibular contouring in Asians. J Craniofac Surg. (2011) 22(1):230–2. 10.1097/SCS.0b013e3181f4af97 21233762

[16] Gui L Yu D Zhang Z Changsheng LV Tang X Zheng Z. Intraoral one-stage curved osteotomy for the prominent mandibular angle: a clinical study of 407 cases. Aesthetic Plast Surg. (2005) 29(6):552–7. 10.1007/s00266-004-0149-7 16158210

[17] Zhang C Teng L Chan FC Xu JJ Lu JJ Xie F Single stage surgery for contouring the prominent mandibular angle with a broad chin deformity: en-bloc mandibular angle-body-chin curved ostectomy (MABCCO) and outer cortex grinding (OCG). J Craniomaxillofac Surg. (2014) 42(7):1225–33. 10.1016/j.jcms.2014.03.004 24754914

[18] Yang DB Park CG. Mandibular contouring surgery for purely aesthetic reasons. Aesthetic Plast Surg. (1991) 15(1):53–60. 10.1007/BF02273834 1994650

[19] Fu X Qiao J Girod S Niu F Liu JF Lee GK Standardized protocol for virtual surgical plan and 3-dimensional surgical template-assisted single-stage mandible contour surgery. Ann Plast Surg. (2017) 79(3):236–42. 10.1097/SAP.0000000000001149 28737554

[20] Zhang C Ma MW Xu JJ Lu JJ Xie F Yang LY Application of the 3D digital ostectomy template (DOT) in mandibular angle ostectomy (MAO). J Craniomaxillofac Surg. (2018) 46(10):1821–7. 10.1016/j.jcms.2018.07.026 30197213

[21] Seok H Kim SG Park YW Lee YC. Postoperative three-dimensional evaluation of mandibular contouring surgery using computer-assisted simulation planning and a three-dimensional-printed surgical guide. J Craniofac Surg. (2017) 28(3):768–70. 10.1097/SCS.0000000000003442 28468162

[22] Qiu X Han W Dai L Zhang Y Zhang J Chai G Assessment of an artificial intelligence mandibular osteotomy design system: a retrospective study. Aesth Plast Surg. (2022) 46(3):1303–13. 10.1007/s00266-021-02698-2 35048148

[23] Straughan DM Yaremchuk MJ. Changing mandible contour using computer designed/computer manufactured alloplastic implants. Aesthet Surg J. (2021) 41(10):NP1265–75. 10.1093/asj/sjab200 33884405

[24] Endara MR Allred LJ Han KD Baker SB. Applications of fat grafting in facial aesthetic skeletal surgery. Aesthet Surg J. (2014) 34(3):363–73. 10.1177/1090820X14525964 24676411

[25] Masden D Baker S. A novel approach for correcting mandibular asymmetry with a combination of autologous fat and alloplastic implants. Aesthet Surg J. (2010) 30(4):513–5. 10.1177/1090820X10380704 20829247

[26] Brandt MG Moore CC. Implants in facial skeletal augmentation. Curr Opin Otolaryngol Head Neck Surg. (2013) 21(4):396–9. 10.1097/MOO.0b013e32836385d1 23799416

[27] Goldsmith D Horowitz A Orentlicher G. Facial skeletal augmentation using custom facial implants. Atlas Oral Maxillofac Surg Clin North Am. (2012) 20(1):119–34. 10.1016/j.cxom.2011.12.002 22365434

[28] Oliver JD Eells AC Saba ES Boczar D Restrepo DJ Huayllani MT Alloplastic facial implants: a systematic review and meta-analysis on outcomes and uses in aesthetic and reconstructive plastic surgery. Aesthetic Plast Surg. (2019) 43(3):625–36. 10.1007/s00266-019-01370-0 30937474

[29] Li S Mi L Bai L Liu Z Li L Wu Y Application of 3D printed titanium mesh and digital guide plate in the repair of mandibular defects using double-layer folded fibula combined with simultaneous implantation. Front Bioeng Biotechnol. (2024) 12:1350227. 10.3389/fbioe.2024.1350227 38456007 PMC10917970

